# FIGHT‐102: A phase 1 study of pemigatinib in Japanese patients with advanced malignancies

**DOI:** 10.1002/cam4.5798

**Published:** 2023-03-31

**Authors:** Yutaka Fujiwara, Yasutoshi Kuboki, Masayuki Furukawa, Nobumasa Mizuno, Hiroki Hara, Tatsuya Ioka, Makoto Ueno, Yasuo Takahashi, Shunji Takahashi, Shinji Takeuchi, Christine Lihou, Tao Ji, Chenwei Tian, Toshio Shimizu

**Affiliations:** ^1^ National Cancer Center Hospital Tokyo Japan; ^2^ Aichi Cancer Center Nagoya Japan; ^3^ National Cancer Central Hospital East Chiba Japan; ^4^ Kyushu Cancer Center Fukuoka Japan; ^5^ Saitama Cancer Center Saitama Japan; ^6^ Department of Oncology Center Yamaguchi University Hospital Ube Japan; ^7^ Kanagawa Cancer Center Yokohama Japan; ^8^ Hokkaido Cancer Center Sapporo Japan; ^9^ Cancer Institute Hospital of JFCR Tokyo Japan; ^10^ Kanazawa University Hospital Kanazawa Japan; ^11^ Incyte Corporation Wilmington Delaware USA; ^12^ Wakayama Medical University Wakayama Japan

**Keywords:** advanced solid tumors, FGFR, Japanese patients, pemigatinib, phase 1 clinical trial

## Abstract

**Background:**

FIGHT‐102 was a phase 1, dose‐escalation, dose‐expansion study of pemigatinib in Japanese patients with advanced solid tumors. Here, we report safety, tolerability, and preliminary efficacy of pemigatinib from FIGHT‐102.

**Methods:**

Patients (≥20 years old) self‐administered oral pemigatinib 9, 13.5, or 18 mg QD on intermittent dosing (Part 1) or 13.5 mg QD intermittent or continuous dosing (Part 2). A dosing cycle was 21 days (2 weeks on/1 week off or 21 continuous days). Primary endpoint was safety. Secondary endpoints were pharmacokinetics, pharmacodynamics, and preliminary efficacy.

**Results:**

Forty‐four patients (Part 1, *n* = 14; Part 2, *n* = 30) were enrolled; most common tumors, cholangiocarcinoma, *n* = 8; esophageal, *n* = 6; 26 patients had confirmed *FGF/FGFR* alterations (Part 1, *n* = 3; Part 2, *n* = 23); 70.5% had ≥3 prior systemic therapies. Maximum tolerated dose was not identified. The recommended phase 2 dosage was determined to be 13.5 mg QD. Most common treatment‐emergent adverse events (TEAEs) were hyperphosphatemia (81.8%), dysgeusia (45.5%), stomatitis (43.2%), and alopecia (38.6%); most frequent Grade ≥3 TEAEs were anemia and decreased appetite (9.1% each). In Part 1, no patient achieved partial response (PR) or complete response, and 7 (50.0%) patients had stable disease (SD). In Part 2, 5 (16.7%) patients achieved PR (one each with cholangiocarcinoma, gall bladder cancer, breast cancer, urothelial tract/bladder cancer, and sweat gland carcinoma) and 6 (20%) had SD. Median duration of response was 9.56 months (95% CI: 4.17, 14.95).

**Conclusions:**

Pemigatinib demonstrated manageable adverse events, consistent pharmacokinetics and pharmacodynamics profiles, and preliminary efficacy in Japanese patients with advanced solid tumors.

## Graphical abstract

FIGHT‐102 (NCT03235570) was an open‐label, phase 1 study of pemigatinib, a potent and selective inhibitor of FGFR1‐3, in Japanese patients with advanced solid malignancies. The safety, pharmacokinetics, and pharmacodynamic profiles of pemigatinib in this study are consistent with those reported in the phase 1 FIGHT‐101 and phase 2 FIGHT‐202 studies. No new safety signals were reported, and promising efficacy was observed with responses across tumor types including cholangiocarcinoma with *FGFR2* alterations.

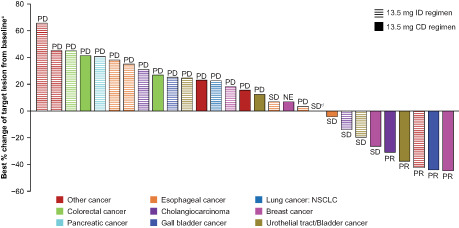

## 1 INTRODUCTION

Fibroblast growth factor receptor (FGFR) signaling regulates essential cellular functions including proliferation and survival, and is mediated by crosstalk between 4 highly conserved tyrosine kinase receptors (FGFR1, FGFR2, FGFR3, and FGFR4) and 22 fibroblast growth factor (FGF) ligands.^1^, ^2^, ^3^ Binding of an FGF results in conformational changes in the FGFR leading to tyrosine kinase activation and subsequent activation of the downstream signaling cascade.^1^

Genetic alterations in *FGFR*, including amplifications, mutations, fusions, or rearrangements, may result in ligand‐independent, constitutive activation of the receptor or aberrant ligand‐dependent signaling and can lead to the establishment and progression of cancer.^2^, ^4^ These alterations occur in a variety of cancers including glioblastoma, breast cancer, lung cancer, bladder cancer, and cholangiocarcinoma.^1^ Some alterations are more frequently observed in certain cancers, for example, *FGFR3* mutations or translocations in bladder cancer, *FGFR2* fusions or rearrangements in cholangiocarcinoma, and *FGFR1* rearrangements in myeloid/lymphoid neoplasms.^5^, ^6^, ^7^, ^8^, ^9^ Strong evidence for the role of the FGFR pathway in tumor proliferation has led to the development of targeted FGFR inhibitors.^2^

Pemigatinib (INCB054828) is a potent and selective inhibitor of *FGFR1*, *FGFR2*, and *FGFR3* ^10^ for the treatment of adults with previously treated, unresectable, locally advanced/metastatic cholangiocarcinoma with an *FGFR2* fusion or other rearrangement.^11^ In the FIGHT‐101 study (NCT02393248), a phase 1/2, dose‐escalation/dose‐expansion study of pemigatinib in patients from the United States and Denmark with refractory advanced malignancies, the recommended dosage for further studies was determined as 13.5 mg QD based on the pharmacologic and safety results.^11^ The study evaluated pemigatinib dosages ranging from 1 to 20 mg QD.^11^ PK analysis showed that for doses of ≥6 mg, *C*_max_ was reached within 1–2 h, with a dose‐independent terminal half‐life of 15 h, supporting QD dosing.^11^ At the recommended dosage of 13.5 mg QD, the geometric mean half‐life was 15.4 h (CV%, 51.6%), steady‐state *C*_max_ was 236 nM (56.4%), and AUC0‐24 was 2620 h × nM (54.1%).^11^ The objective response rate (ORR) was 9.4% (95% confidence interval [CI], 4.9–15.8), including 12 partial responses (PRs) across tumor types.^11^ The ORR was highest for patients with *FGFR* fusions or rearrangements (25.0% [95% CI, 8.7–49.1]). The median duration of response (DOR) for all responders was 7.3 months (95% CI, 3.3–14.5). The median progression‐free survival (PFS) was 5.7 months (95% CI, 2.8–10.0) in patients with *FGFR* fusions or rearrangements.

Based on promising safety and efficacy results from FIGHT‐101, the pivotal phase 2 FIGHT‐202 study (NCT02924376) was initiated. In FIGHT‐202, pemigatinib demonstrated improved and sustained responses in patients with advanced or metastatic cholangiocarcinoma with *FGFR2* fusions or rearrangements, with an ORR of 37.0% (95% CI, 27.9–46.9), median DOR of 8.1 months (95% CI, 5.7–13.1), median PFS of 7.0 months (95% CI, 6.1–10.5), and an estimated median overall survival of 17.5 months (95% CI, 14.4–22.9).^12^ Based on these results, pemigatinib was approved in several regions and countries, including Japan for the treatment of patients with previously treated unresectable, locally advanced, or metastatic cholangiocarcinoma with an *FGFR2* fusion or other rearrangement.^13^, ^14^, ^15^, ^16^

The clinical benefit observed in patients in FIGHT‐101 prompted the FIGHT‐102 study (NCT03235570), an open‐label, phase 1 study of pemigatinib in Japanese patients with advanced solid malignancies. Here, we report the safety, tolerability, pharmacokinetics (PK), pharmacodynamics, and preliminary efficacy of pemigatinib in FIGHT‐102.

## 2 METHODS

### 2.1 Study design and objectives

FIGHT‐102 was conducted in 2 parts (Figure S1). Part 1 examined dose escalation and used a standard 3 + 3 design to evaluate the safety and pharmacological activity of pemigatinib. Part 2 (dose expansion) further evaluated the safety and preliminary efficacy of pemigatinib at the recommended phase 2 dose (RP2D) determined in Part 1. Patients continued treatment as long as it provided benefit and they did not meet any criteria for study withdrawal. The safety follow‐up period was 30 (+5) days after treatment. Patients who discontinued for a reason other than disease progression were followed up every 9 weeks for disease status. Once disease progression was confirmed or a new therapy was initiated, patients were assessed every 12 weeks for survival.

Primary objectives were to evaluate the safety, tolerability, and dose‐limiting toxicity (DLTs) and to determine the maximum tolerated dose (MTD) and/or the RP2D of pemigatinib. Secondary objectives were to evaluate pemigatinib PK and pharmacodynamics and to assess the preliminary efficacy by ORR in patients with measurable disease. Exploratory objective included DOR.

### 2.2 Study treatment

In Part 1, patients self‐administered oral pemigatinib starting with 9 mg once daily (QD) on a 2‐weeks‐on/1 week‐off intermittent dosing (ID) regimen. The safety and tolerability of each dose regimen was observed for 21 days (1 cycle) before escalation to the next dose. Dose increases were limited to ≤50% in successive cohorts. MTD was defined as the maximum dose at which one‐third or fewer of patients reported a DLT. The pharmacologically active dose was defined as the one at which at least 80% of participants developed hyperphosphatemia (defined as serum phosphorus ≥5.5 mg/dL). In Part 2, patients started at the RP2D determined in Part 1.

A protocol amendment (November 6, 2018) introduced a continuous dosing (CD) regimen of pemigatinib 13.5 mg and the possibility of up‐titration for patients receiving 13.5 mg (ID or CD) to 18 mg beginning at Cycle 2 Day 1. At the time of protocol development for this study, the FIGHT‐101 study of pemigatinib at doses ranging from 1 to 20 mg QD was ongoing in the United States and Denmark. Interim safety data from FIGHT‐101 accrued at that time of protocol development for FIGHT‐102 supported an ID regimen starting at 9 mg QD and introduction of CD with a starting dose of 13.5 mg.

### 2.3 Study conduct

The protocol was approved by the Institutional Review Board or independent ethics committee of each study center. All patients provided written informed consent. The study was conducted in accordance with the International Council for Harmonisation Guideline for Good Clinical Practice, the ethical principles of the Declaration of Helsinki, Japan Good Clinical Practices, and other applicable local regulatory requirements.

### 2.4 Patients

Eligible male or female Japanese patients ≥20 years of age were enrolled in the study. Part 1 enrolled patients with any histologically confirmed, measurable advanced solid tumor malignancy; Part 2 enrolled patients with any histologically confirmed, measurable advanced solid tumor malignancy and any documented *FGF/FGFR* alteration. *FGF/FGFR* status was assessed based on local laboratory results and retrospectively confirmed by a central laboratory. At the discretion of the investigator, patients could be enrolled based on local laboratory results that were not confirmed by a central laboratory. Patients had advanced or metastatic and recurrent cancer that had progressed following at least one course of therapy and for which an appropriate treatment option was not available. They had recovered from adverse events (AEs; ≤Grade 1 at baseline) due to previously administered therapies and were expected to live >12 weeks at the time of screening. Patients had an Eastern Cooperative Oncology Group performance status (ECOG PS) of ≤1 in Part 1 and ≤2 in Part 2. Genomic testing was mandatory for all enrolled patients. Therefore, patients must have been willing to undergo a pretreatment tumor biopsy or able to provide an archival tumor sample no more than 2 years old.

Patients were excluded from the study if they had received: a selective FGFR inhibitor ever, any anticancer medications or any other investigational drug within 21 days or 5 half‐lives (whichever is longer; 6 weeks for mitomycin‐C or nitrosoureas, 7 days for tyrosine kinase inhibitors), any potent *CYP3A4* inhibitor or inducer within 14 days or 5 half‐lives (whichever is shorter), or radiotherapy within 2 weeks before first dose of study drug. Patients were also excluded if they had hemoglobin ≤8.5 g/dL, platelets ≤75 × 10^9^ cells/L, absolute neutrophil count ≤1.0 × 10^9^ cells/L, total bilirubin ≥1.5 × institutional upper limit of normal (ULN), aspartate aminotransferase (AST) or alanine aminotransferase (ALT) ≥3× ULN, alkaline phosphatase ≥2.5× ULN, creatinine clearance ≤50 mL/min, parathyroid hormone >1.5× ULN, serum phosphorous above ULN, or serum calcium or serum‐albumin calcium outside of institutional normal range. Additional exclusion criteria included history and/or current evidence of ectopic mineralization/calcification, current evidence of corneal disorder/keratopathy, untreated brain or CNS metastases or brain/CNS metastases that have progressed, HIV infection, history of clinically significant or uncontrolled cardiac disease requiring therapy, or a chronic or currently active infectious disease requiring systemic treatment.

### 2.5 Assessments

#### 2.5.1 Safety

Safety and tolerability were assessed by monitoring the frequency and duration of AEs, measuring vital signs, conducting 12‐lead electrocardiograms, and performing comprehensive eye examinations and physical examinations. Eye examinations were performed once every 3 cycles ±14 days and as clinically indicated, starting on Cycle 3. Severity of AEs was assessed using the National Cancer Institute Common Terminology Criteria for Adverse Events (NCI CTCAE) version 4.03 Grades 1 through 4. Severity of hyperphosphatemia is not included in CTCAE version 4.03 and was graded as follows: Grade 1 is asymptomatic or with mild symptoms requiring clinical or diagnostic observations only; Grade 2 requires minimal, local, or noninvasive intervention, and limits age‐appropriate activities of daily living; Grade 3 is severe or medically significant hyperphosphatemia that is not immediately life‐threatening, for which hospitalization or prolongation of hospitalization is indicated; Grade 4 hyperphosphatemia requires urgent intervention and is associated with life‐threatening consequences.

#### 2.5.2 PK and pharmacodynamic analysis

Blood samples for pemigatinib PK assessments were collected during both ID and CD regimens before dosing on Days 1, 2, 8, 14, 15, and 16 of Cycle 1. Postdose samples were collected at 0.5, 1, 2, 4, 6, and 8 h on Day 1, and at 1 h on Day 14 of Cycle 1. For patients in the CD regimen, a postdose sample at 6 h on Day 14 was also collected. Pemigatinib plasma concentrations were determined using bioanalytical methods previously described by Ji et al.^17^, ^18^

Plasma samples for pharmacodynamic assessments were collected predose on Days 1, 14, and 15 of Cycle 1 and any time on Day 1 (±3 days) of the subsequent cycles. Blood samples for evaluating comprehensive serum chemistry including serum phosphate concentrations after treatment of pemigatinib were collected on Days 1, 8, and 15 of Cycle 1, and Day 1 of each cycle thereafter. Baseline FGF23 concentrations were assessed in plasma samples collected on Days 1 and 15 of Cycle 1 using a commercially available assay (Meso Scale Discovery). The pharmacodynamic effects of pemigatinib were determined in plasma from samples collected predose and at Cycle 1 Day 15 (0 h) before pemigatinib administration. Inhibition of FGFR2 was assessed using an ex vivo assay that measured phosphorylated *FGFR2α* concentration following exposure of *FGFR2*‐amplified gastric cancer cells (KATOIII) to patient plasma.

#### 2.5.3 Efficacy

Efficacy assessments occurred at screening (baseline scan) and on Day 15 of every third cycle with a ±2‐day window. Tumor status was assessed by the investigator using appropriate disease‐specific techniques. For solid tumors, the Response Evaluation Criteria in Solid Tumors (RECIST) 1.1 criteria were used,^19^ and the recommended method for measuring tumor burden was computed tomography scan. Alternative modalities compatible with RECIST 1.1 could be used at the investigator's discretion provided they were used consistently throughout the study.

### 2.6 Statistical analysis

No calculation of statistical power was performed for this study because no analyses of clinical significance were planned. Safety data (vital signs, ECGs, routine laboratory tests, physical examinations, and AEs) were summarized descriptively. Plasma pemigatinib concentrations and PK parameters (maximum observed plasma concentration [*C*_max_], time to reach *C*_max_ [*t*_max_], *C*_min_, area under the plasma concentration‐time curve from *t* = 0 to the last measurable concentration at time t [AUC_0−*t*_], area under the steady‐state plasma or serum concentration‐time curve over 1 dose interval [AUC_0‐*τ*_], terminal half‐life (*t*_½_), and oral clearance [CL/F]) were calculated using standard noncompartmental (model‐independent) methods and summarized for the PK/pharmacodynamic population. Plasma concentration data from ID and CD administration were pooled for each dosage for PK analysis because no changes in PK parameters with CD dosing were expected after steady‐state concentrations were reached at approximately Day 4. Pharmacodynamic data were presented using summary statistics.

Both the efficacy and the safety population included all patients who received at least 1 dose of study drug. The PK/pharmacodynamic population included all patients who received at least one dose of study drug and provided at least one postdose plasma sample for PK/pharmacodynamic measurement. No comparisons are made between patients or against historical controls; only comparisons to pretreatment conditions were made.

## 3 RESULTS

### 3.1 Patients

**FIGURE 1:**
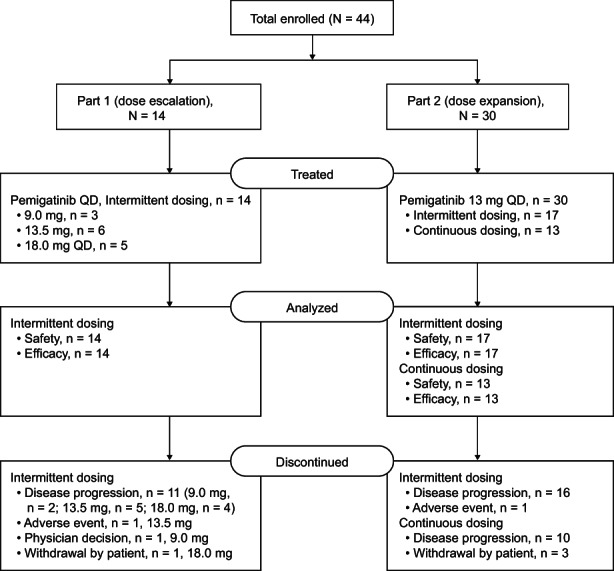
Summary of patient disposition. QD, once daily.

A total of 44 patients with advanced solid tumors were enrolled at 10 study centers in Japan: 14 in Part 1 (dose escalation) and 30 in Part 2 (dose expansion). Patients received pemigatinib 9, 13.5, or 18 mg on an ID regimen in Part 1 and pemigatinib 13.5 mg on ID or CD in Part 2 (Figure 1). All 44 patients discontinued pemigatinib. Progressive disease (PD; 84.1%) was the most common reason for treatment discontinuation. Death (65.9%) was the most common reason for study withdrawal.

Patients were predominantly male (61.4%), with median age of 63 and most (75.0%) had an ECOG PS of 0 (Table 1). Most patients (70.5%) had received ≥3 prior systemic therapies. The most common tumor types were cholangiocarcinoma (*n* = 8, 18.2% [cholangiocarcinoma, *n* = 4; “Other intrahepatic cholangiocarcinoma”, *n* = 4]) and esophageal cancer (*n* = 6, 13.6%). An additional patient was reported as having lower bile duct cancer. Twenty‐six patients had centrally confirmed *FGF/FGFR* alterations (Part 1, *n* = 3; Part 2, *n* = 23) as shown in Table S1. Two patients in Part 1 and seven in Part 2 had locally confirmed alterations only. Patients completed a mean 4.5 cycles of pemigatinib. The median duration of treatment across all dosages was 56.0 days (range, 6–496). In Part 1, the median duration of exposure was 58.0 days (range, 9–254). In Part 2, the median duration of exposure for the ID regimen was slightly longer (56.0 days [range, 6–496]) compared with the CD regimen (45.0 days [range, 19–309]).

**TABLE 1:** Baseline demographics and disease characteristics.

Variable	Pemigatinib intermittent dosing				Pemigatinib continuous dosing 13.5 mg (*n* = 13)	Total (*n* = 44)
	9 mg (*n* = 3)	13.5 mg (*n* = 23)	18 mg (*n* = 5)	Subtotal (*n* = 31)		
Median age, years (range)	58.0 (43–60)	64.0 (32–79)	64.0 (54–70)	64.0 (32–79)	63.0 (47–69)	63.0 (32–79)
Sex, *n* (%)						
Men	2 (66.7)	14 (60.9)	4 (80.0)	20 (64.5)	7 (53.8)	27 (61.4)
Women	1 (33.3)	9 (39.1)	1 (20.0)	11 (35.5)	6 (46.2)	17 (38.6)
Median body weight, kg (range)	60.4 (42.0–76.8)	59.3 (39.8–87.6)	55 (49.7–63.1)	58.8 (39.8–87.6)	57.4 (43.7–98.0)	57.6 (39.8–98.0)
ECOG status, *n* (%)						
0	3 (100.0)	17 (73.9)	4 (80.0)	24 (77.4)	9 (69.2)	33 (75.0)
1	0	5 (21.7)	1 (20.0)	6 (19.4)	4 (30.8)	10 (22.7)
2	0	1 (4.3)	0	1 (3.2)	0	1 (2.3)
Solid tumor type, *n* (%)						
Breast cancer	0	2 (8.7)	0	2 (6.5)	3 (23.1)	5 (11.4)
Cholangiocarcinoma^a^	0	3 (13.0)	0	3 (9.7)	1 (7.7)	4 (9.1)
Colorectal cancer	0	2 (8.7)	0	2 (6.5)	2 (15.4)	4 (9.1)
Esophageal cancer	0	5 (21.7)	0	5 (16.1)	1 (7.7)	6 (13.6)
Gallbladder cancer	0	1 (4.3)	0	1 (3.2)	1 (7.7)	2 (4.5)
Gastric cancer	1 (33.3)	0	0	1 (3.2)	0	1 (2.3)
NSCLC	0	2 (8.7)	0	2 (6.5)	0	2 (4.5)
Neuroendocrine cancer	0	0	1 (20.0)	1 (3.2)	0	1 (2.3)
Pancreatic cancer: neuroendocrine	0	1 (4.3)	0	1 (3.2)	0	1 (2.3)
Urothelial tract/bladder cancer: renal pelvis	0	1 (4.3)	0	1 (3.2)	1 (7.7)	2 (4.5)
Urothelial tract/bladder cancer: bladder	0	1 (4.3)	0	1 (3.2)	1 (7.7)	2 (4.5)
Other^a^	2 (66.7)	5 (21.7)	4 (80.0)	11 (35.5)	3 (23.1)	14 (31.8)
Median number of prior systemic therapies (range)	1 (1–1)	4 (0–9)	4 (3–4)	3 (0–9)	4 (1–14)	4 (0–14)
Centrally confirmed *FGF/FGFR* status at baseline, *n* (%)^b^						
Alterations detected	0	18 (78.3)	0	18 (58.1)	8 (61.5)	26 (59.1)
Not detected	0	2 (8.7)	2 (40.0)	4 (12.9)	2 (15.4)	6 (13.6)
Missing or not assessed	3 (100)	3 (13.0)	3 (60.0)	9 (29.0)	3 (23.1)	12 (27.3)

*Note*: Missing indicates that this information is not available.

Abbreviations: ECOG, Eastern Cooperative Oncology Group; FGF, fibroblast growth factor; FGFR, FGF receptor; NSCLC, nonsmall cell lung cancer.

^a^ Four patients were categorized as having a tumor type of “other: intrahepatic cholangiocarcinoma” and 1 was categorized as having lower bile duct cancer. These five patients were included in the “other solid tumor” category.

^b^ Seven patients enrolled in Part 2 did not have *FGF/FGFR* alterations detected by the central laboratory but did have *FGF/FGFR* alteration(s) detected by a local laboratory at baseline and were permitted to continue treatment based on investigator's discretion. See Table S1 for full details of centrally confirmed *FGF/FGFR* status.

### 3.2 Safety

No DLTs were reported in Part 1 of the study and the MTD of pemigatinib was not reached. Treatment‐emergent adverse events (TEAEs) were experienced by all enrolled patients (*N* = 44); The most common TEAEs were hyperphosphatemia (overall, 81.8%; ID, 80.6%; CD, 84.6%), dysgeusia (overall, 45.5%; ID, 35.5%; CD, 69.2%), stomatitis (overall, 43.2%; ID, 29.0%; CD, 76.9%), and alopecia (overall, 38.6%; ID, 32.3%; CD, 53.8%) (Table 2). All hyperphosphatemia events were Grades 1 or 2. Grade ≥3 TEAEs occurred in 21 patients (15 [48.4%] in ID and 6 [46.2%] in CD). The most common Grade ≥3 TEAEs were anemia (9.1%; ID, 12.9%; CD, 0%) and decreased appetite (9.1%; ID, 6.5%; CD, 15.4%).

**TABLE 2:** Common TEAEs (>5%) and clinically notable TEAEs.

Number of events (%)	Pemigatinib intermittent dosing								Pemigatinib continuous dosing		Total (*n* = 44)	
	9 mg (*n* = 3)		13.5 mg (*n* = 23)		18 mg (*n* = 5)		Subtotal (*n* = 31)		13.5 mg (*n* = 13)			
	All grades	Grade ≥3	All grades	Grade ≥3	All grades	Grade ≥3	All grades	Grade ≥3	All grades	Grade ≥3	All grades	Grade ≥3
Hyperphosphatemia	2 (66.7)	0	18 (78.3)	0	5 (100.0)	0	25 (80.6)	0	11 (84.6)	0	36 (81.8)	0
Dysgeusia	1 (33.3)	0	8 (34.8)	0	2 (40.0)	0	11 (35.5)	0	9 (69.2)	0	20 (45.5)	0
Stomatitis	1 (33.3)	0	4 (17.4)	0	4 (80.0)	0	9 (29.0)	0	10 (76.9)	1 (7.7)	19 (43.2)	1 (2.3)
Alopecia	1 (33.3)	0	7 (30.4)	0	2 (40.0)	0	10 (32.3)	0	7 (53.8)	0	17 (38.6)	0
Nausea	1 (33.3)	0	7 (30.4)	1 (4.3)	3 (60.0)	0	11 (35.5)	1 (3.2)	5 (38.5)	0	16 (36.4)	1 (2.3)
Diarrhea	1 (33.3)	0	6 (26.1)	0	1 (20.0)	0	8 (25.8)	0	8 (61.5)	1 (7.7)	16 (36.4)	1 (2.3)
Decreased appetite	1 (33.3)	1 (33.3)	7 (30.4)	1 (4.3)	3 (60.0)	0	11 (35.5)	2 (6.5)	5 (38.5)	2 (15.4)	16 (36.4)	4 (9.1)
Constipation	1 (33.3)	0	7 (30.4)	0	2 (40.0)	0	10 (32.3)	0	4 (30.8)	1 (7.7)	14 (31.8)	1 (2.3)
Arthralgia	0	0	3 (13.0)	0	1 (20.0)	0	4 (12.9)	0	3 (23.1)	0	7 (15.9)	0
AST increased	1 (33.3)	0	4 (17.4)	0	0	0	5 (16.1)	0	2 (15.4)	1 (7.7)	7 (15.9)	1 (2.3)
Anemia	1 (33.3)	1 (33.3)	1 (4.3)	1 (4.3)	2 (40)	2 (40.0)	4 (12.9)	4 (12.9)	3 (23.1)	0	7 (15.9)	4 (9.1)
Fatigue	0	0	3 (13.0)	0	1 (20)	0	4 (12.9)	0	3 (23.1)	0	7 (15.9)	0
Blood creatinine increased	0	0	4 (17.4)	0	1 (20.0)	0	5 (16.1)	0	1 (7.7)	0	6 (13.6)	0
Paronychia	0	0	2 (8.7)	0	0	0	2 (6.5)	0	4 (30.8)	0	6 (13.6)	0
ALT increased	2 (66.7)	0	2 (8.7)	0	0	0	4 (12.9)	0	2 (15.4)	1 (7.7)	6 (13.6)	1 (2.3)
Malaise	0	0	2 (8.7)	0	0	0	2 (6.5)	0	4 (30.8)	0	6 (13.6)	0
Vomiting	2 (66.7)	0	4 (17.4)	0	0	0	6 (19.4)	0	0	0	6 (13.6)	0
Dry mouth	0	0	1 (4.3)	0	0	0	1 (3.2)	0	4 (30.8)	0	5 (11.4)	0
Keratitis	1 (33.3)	0	2 (8.7)	0	0	0	3 (9.7)	0	2 (15.4)	0	5 (11.4)	0
Onychomadesis	0	0	2 (8.7)	0	0	0	2 (6.5)	0	3 (23.1)	0	5 (11.4)	0
Palmar‐plantar erythrodysesthesia syndrome	0	0	2 (8.7)	0	0	0	2 (6.5)	0	3 (23.1)	0	5 (11.4)	0
Serous retinal detachment	1 (33.3)	0	3 (13.0)	0	0	0	4 (12.9)	0	1 (7.7)	0	5 (11.4)	0
Trichiasis	0	0	2 (8.7)	0	0	0	2 (6.5)	0	2 (15.4)	0	4 (9.1)	0
Blood alkaline phosphatase increased	0	0	2 (8.7)	0	0	0	2 (6.5)	0	2 (15.4)	1 (7.7)	4 (9.1)	1 (2.3)
Back pain	0	0	1 (4.3)	0	0	0	1 (3.2)	0	3 (23.1)	0	4 (9.1)	0
Tumor pain	0	0	3 (13.0)	1 (4.3)	1 (20.0)	1 (20.0)	4 (12.9)	2 (6.5)	0	0	4 (9.1)	2 (4.5)
Cough	0	0	2 (8.7)	0	1 (20.0)	0	3 (9.7)	0	1 (7.7)	0	4 (9.1)	0
Dry skin	0	0	1 (4.3)	0	1 (20.0)	0	2 (6.5)	0	2 (15.4)	0	4 (9.1)	0
Dry eye	0	0	2 (8.7)	0	0	0	2 (6.5)	0	1 (7.7)	0	3 (6.8)	0
Punctate keratitis	0	0	1 (4.3)	1 (4.3)	0	0	1 (3.2)	1 (3.2)	2 (15.4)	0	3 (6.8)	1 (2.3)
Pyrexia	0	0	3 (13.0)	1 (4.3)	0	0	3 (9.7)	1 (3.2)	0	0	3 (6.8)	1 (2.3)
Conjunctivitis	0	0	1 (4.3)	0	0	0	1 (3.2)	0	2 (15.4)	0	3 (6.8)	0
WBC count decreased	0	0	0	0	0	0	0	0	3 (23.1)	0	3 (6.8)	0
Myalgia	1 (33.3)	0	1 (4.3)	0	0	0	2 (6.5)	0	1 (7.7)	0	3 (6.8)	0
Insomnia	0	0	2 (8.7)	0	0	0	2 (6.5)	0	1 (7.7)	0	3 (6.8)	0
Epistaxis	0	0	0	0	0	0	0	0	3 (23.1)	0	3 (6.8)	0
Nail discoloration	0	0	1 (4.3)	0	0	0	1 (3.2)	0	2 (15.4)	0	3 (6.8)	0
Gastritis	0	0	2 (8.7)	0	0	0	2 (6.5)	0	1 (7.7)	0	3 (6.8)	0
Clinically notable TEAEs												
Hyperphosphatemia	3 (100.0)	0	18 (78.3)	0	5 (100)	0	26 (83.9)	0	11 (84.6)	0	37 (84.1)	0
Hyperphosphatemia												
Blood phosphorus increased												
Nail toxicity	1 (33.3)	0	5 (21.7)	0	0	0	6 (19.4)	0	8 (61.5)	0	14 (31.8)	0
Paronychia												
Onychomadesis												
Nail discoloration												
Onycholysis												
Nail ridging												
Dry eye	1 (33.3)	0	6 (26.1)	1 (4.3)	0	0	7 (22.6)	1 (3.2)	4 (30.8)	0	11 (25.0)	1 (2.3)
Dry eye												
Keratitis												
Lacrimation increased												
Punctate keratitis												
Serous retinal detachment	1 (33.3)	0	6 (26.1)	0	0	0	7 (22.6)	0	1 (7.7)	0	8 (18.2)	0
Serous retinal detachment												
Subretinal fluid												
Chorioretinopathy												
Eyelash changes	0	0	2 (8.7)	0	0	0	2 (6.5)	0	2 (15.4)	0	4 (9.1)	0
Trichiasis												
Vision blurred	0	0	1 (4.3)	0	1 (20.0)	0	2 (6.5)	0	0	0	2 (4.5)	0
Hypophosphatemia	0	0	1 (4.3)	0	0	0	1 (3.2)	0	1 (7.7)	0	2 (4.5)	0

*Note*: Grade ≥3 TEAEs data are only shown for the all‐grade events presented in the table. Grade ≥3 TEAEs for other events are not presented in the table.

Abbreviations: ALT, alanine transaminase; AST, aspartate transaminase; TEAE, treatment‐emergent adverse event; WBC, white blood cell.

In general, the incidence of individual TEAEs was higher in the CD regimen as compared with ID (Table 2). TEAEs rates that were 20% greater in the CD versus ID regimens were dysgeusia (69.2% vs. 35.5%), stomatitis (76.9% vs. 29.0%), alopecia (53.8% vs. 32.3%), diarrhea (61.5% vs. 25.8%), malaise (30.8% vs. 6.5%), paronychia (30.8% vs. 6.5%), dry mouth (30.8% vs. 3.2%), white blood cell count decreased (23.1% vs. 0%), and epistaxis (23.1% vs. 0%) (Table 2). A similar pattern was seen at the RP2D of 13.5 mg QD with a higher incidence of TEAEs in the CD regimen versus the ID regimen (Table 2).

The majority of patients (97.7%) experienced at least 1 treatment‐related TEAE. The most common treatment‐related TEAEs were hyperphosphatemia (overall, 81.8%; ID, 80.6%; CD, 84.6%), dysgeusia (overall, 40.9%; ID, 32.3%; CD, 61.5%), stomatitis (overall, 40.9%; ID, 25.8%; CD, 76.9%), alopecia (overall, 38.6%; ID, 32.3%; CD, 53.8%), and nausea (overall, 31.8%; ID, 32.3%; CD, 30.8%) (Table S2). Eight treatment‐related TEAEs of Grade ≥3 severity occurred in 7/44 (15.9%) patients: 1 instance (2.3%) each of anemia, punctate keratitis, diarrhea, stomatitis, ALT increased, AST increased, decreased appetite, and hematuria. Similar to the pattern for overall TEAEs, the incidence of treatment‐related TEAEs was generally higher among patients who received pemigatinib as a CD regimen as compared with ID.

Overall, 22 patients (50%; 12 [38.7%] in ID and 10 [76.9%] in CD) had a TEAE leading to dose interruption. Six patients (13.6%; 5 [16.1%] in ID and 1 [7.7%] in CD) had a TEAE leading to dose reduction. Four patients (9.1%; all in ID [12.9%]) had a TEAE leading to pemigatinib discontinuation. The most common TEAE leading to dose interruption was hyperphosphatemia (15.9%; ID, 9.7%; CD, 30.8%). No patients discontinued pemigatinib treatment due to hyperphosphatemia. The most common TEAE leading to dose reduction was fatigue (overall, 4.5%; ID, 6.5%; CD, 0%). All TEAEs leading to dose reduction were considered related to pemigatinib, except for 1 Grade 2 event of fatigue. All TEAEs leading to treatment discontinuation occurred with ID (13.5 mg QD) and included ascites, metastatic brain cancer, malignant neoplasm progression, and hyperesthesia in one patient (2.3%) each. Of these, only hyperesthesia (Grade 1) was considered related to pemigatinib.

Serious TEAEs occurred in 16 patients (14 [45.2%] in ID and 2 [15.4%] in CD). Three (6.8%) of these patients had a TEAE with a fatal outcome: malignant neoplasm progression in two patients and dyspnea in one patient. Each of these events were considered unrelated to pemigatinib. All three patients had discontinued treatment attributable to progressive disease. Cholangitis, decreased appetite, and malignant neoplasm progression (in two patients each) were the only serious TEAEs reported in more than one patient.

Clinically notable TEAEs occurred in 88.6% of patients (ID, 90.3%; CD, 84.6%) (Table 2). Pemigatinib dose interruptions due to clinically notable TEAEs occurred in 10 (22.7%) patients. Dose reductions due to clinically notable TEAEs occurred in 3 (6.8%) patients.

Hyperphosphatemia or “blood phosphorous increased” was reported in 37 (84.1%) patients (26 patients in the ID and 11 in the CD regimen). None of the events were Grade ≥3. Hyperphosphatemia led to dose interruption in 7 (15.9%) patients and dose reduction in 1 (2.3%) patient. Hypophosphatemia was reported in 2 (4.5%) patients, 1 each in 13.5 mg ID and 13.5 mg CD regimens. Both events were Grade 2 in severity. The hypophosphatemia event in the ID regimen was considered treatment‐related. None of the hypophosphatemia events led to pemigatinib dose interruption or reduction. Notably, half of patients in the study received lanthanum carbonate, a phosphate‐binding agent for the treatment of hyperphosphatemia (including prophylaxis).

Clinically notable ocular TEAEs included dry eye in 11 (25%) patients (comprised of dry eye: 3 [6.8%]), keratitis: 5 [11.4%], punctate keratitis: 3 [6.8%], and lacrimation increased: 1 [2.3%] as well as serous retinal detachment in 8 (18.2%) patients (including serous retinal detachment: 5 [11.4%], subretinal fluid: 2 [4.5%], and chorioretinopathy: 1 [2.3%]; Table 2). Other clinically notable ocular TEAEs were eyelash changes (trichiasis) in 4 (9.1%) patients and vision blurred in 2 (4.5%). Keratitis and serous retinal detachment led to pemigatinib dose interruption in two patients each. Punctate keratitis led to dose interruption in one patient and to dose reduction in one patient. Grade ≥3 TEAE, punctate keratitis occurred in only one patient receiving 13.5‐mg ID, which improved to Grade 1 after treatment interruption and dose reduction. Nail toxicity TEAEs occurred in 14 (31.8) patients and included paronychia in 6 (13.6%) patients, onychomadesis in 5 (11.4%), nail discoloration in 3 (6.8%), oncholysis in 2 (4.5%), and nail ridging in 1 (2.3%) (Table 2). Oncholysis, onychomadesis, and paronychia led to pemigatinib dose interruption in one patient each. Onycholysis was managed with dose reduction in one patient.

### 3.3 PK and Pharmacodynamics

**FIGURE 2:**
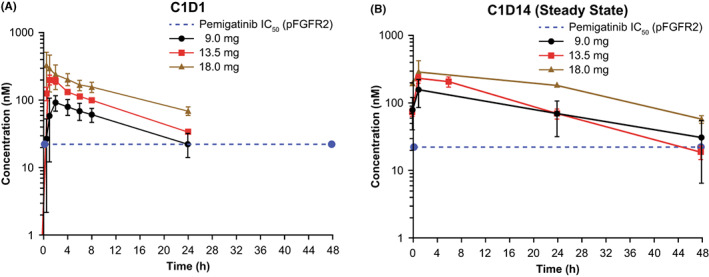
Plasma pemigatinib concentrations on (A) Cycle 1 Day 1 (C1D1), (B) Cycle 1 Day 14 (C1D14; steady state). Data are presented as mean ± SE. FGFR2, fibroblast growth factor receptor 2; IC_50_, concentration that inhibits 50%; pFGFR2, phospho‐FGFR2; SE, standard error.

Blood samples from all 44 patients drawn on Cycle 1 Day 1 (C1D1) and from 39 patients drawn on C1D14 were used to assess pemigatinib PK. The geometric mean (CV%) peak plasma concentration (*C*_max_) of 216 nM (77.0) was attained in median time (*t*max) of 1 h after dosing with pemigatinib 13.5 mg (Figure 2, Table S3). After a single dose of pemigatinib 9 or 13.5 mg, plasma concentrations of pemigatinib remained at or above the in vivo concentration that inhibits 50% (IC_50_; 22.6 nM) for the inhibition of pFGFR2 for a full 24 h (Figure 2).^10^ The steady‐state geometric mean half‐life (*t*_1/2_) was 13.6 h (Table S4).

The geometric mean accumulation ratio for area under the plasma concentration‐time curve from 0 to 24 h (AUC_0‐24_) for patients receiving 13.5 mg pemigatinib was 1.43 (Table S4). The geometric mean (CV%) *C*_max_ and AUC_0‐24_ at steady state were 195 nM (77.8%) and 2720 h × nM (55.5%), respectively (Table S4). At steady state, pemigatinib exhibited low oral clearance (geometric mean CL_ss_/F of 10.2 L/h) and moderate volume of distribution (geometric mean *V*_z_/*F* of 201 L; Table S4). An exploratory urine PK analysis showed the geometric mean fraction of pemigatinib dose excreted in urine was 2.48% with a geometric mean renal clearance of 0.240 L/h.

A comparison of pemigatinib PK between the Japanese patients in this study and the predominantly white patients from the FIGHT‐101 study showed similar pemigatinib exposures. With pemigatinib 13.5 mg QD dosing, the steady‐state *C*_max_ geometric means were 195 and 236 nM, and AUC_0‐24h_ geometric means were 2720 h × nM and 2620 h × nM in FIGHT‐102 and FIGHT‐101, respectively (Figure S2).

FGF23 concentrations at baseline and at Day 15 of treatment Cycle 1 were measured in plasma from 41 patients (9 mg, *n* = 3; 13.5 mg, *n* = 34; 18 mg, *n* = 4). Elevated mean FGF23 concentrations at Day 15 of Cycle 1were observed in in 90% (37/41) of these patients. Mean FGF23 concentration increased significantly from 198 ± 29 pg/mL at baseline to 464 ± 49 pg/mL at C1D15 (paired *t*‐test, *p* < 0.05). Induction of plasma FGF23 was observed at all pemigatinib doses with mean increases of 2.6‐, 2.9‐, and 3.9‐fold during treatment with pemigatinib 9, 13.5, and 18 mg, respectively. In patients treated with pemigatinib 13.5 mg QD, median FGF23 concentration rose slightly more than twofold from baseline in the first 2 weeks of treatment, and did not change statistically significantly thereafter (Figure S3).

Ex vivo target inhibition as defined by ≥50% inhibition of pFGFR2α at any timepoint was observed for all patients. Mean inhibition of pFGFR2α at trough pemigatinib concentration (C1D15, 0 h) was 80% (Figure S4).

### 3.4 Efficacy

**FIGURE 3:**
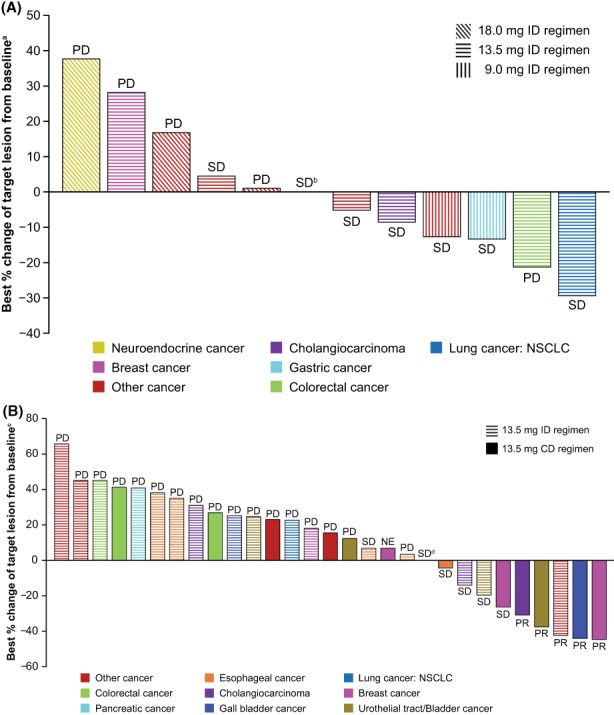
Best percentage change from baseline in target lesion size for patients in (A) Part 1 and (B) Part 2. Bar plots indicate percentage change in target lesion size and colors indicate the present tumor types. Corresponding best overall responses per Response Evaluation Criteria in Solid Tumors v1.1 are also presented. ^a^Assessments were missing for one patient each in 9.0‐ and 18.0‐mg ID regimens, respectively. ^b^Patient had other cancer and received pemigatinib 18.0 mg in ID regimen. ^c^Assessment was missing for 1 patient in the 13.5‐mg ID regimen. ^d^Patient had other cancer and received pemigatinib 13.5 mg in CD regimen. Assessments were missing for two patients in Part 1 and one patient in Part 2. CD, continuous dosing; ID, intermittent dosing; NE, not evaluable; PD, progressive disease; PR, partial response; SD, stable disease.

In Part 1 of the study, no patient had a best overall response (BOR) of complete response or PR, seven patients (50.0%) had a BOR of stable disease (SD). The median best percentage change from baseline in target lesion size was −2.6%. In Part 2, five patients (16.7%) achieved PR and 6 (20%) had SD. Median DOR among responders was 9.56 months (95% CI: 4.17, 14.95). The median of best percentage change from baseline in target lesion size was 15.2%. Best percentage changes in target lesion size for patients in Parts 1 and 2 are shown in Figure 3.

Responses by genomic alterations for all patients in Parts 1 and 2 are shown in Table S5. Five patients in Part 2 achieved a PR while receiving pemigatinib 13.5 mg treatment (ID, *n* = 1; CD, *n* = 4) for the following tumors: cholangiocarcinoma with a locally identified *FGFR2* translocation; gall bladder cancer with *FGF3/4/19* amplification; breast cancer with *FGFR2* amplification; urothelial tract/bladder cancer with *FGF3/4/19* amplification and *FGFR3* alteration; and “other” (apocrine sweat gland carcinoma) with *FGFR2* amplification. Four patients with cholangiocarcinoma had SD; of whom one had a centrally confirmed *FGF1* and *FGFR2* amplification and one had a centrally confirmed *FGFR2* rearrangement and locally identified *FGFR2* translocation. A further three patients with cholangiocarcinoma had BORs of PD. One patient with lower bile duct cancer had SD. Of the two patients with gallbladder cancer, one had a BOR of PR as described above and the other had PD. Among patients with other tumor types who had genomic alterations, SD was attained by one patient with esophageal cancer and centrally assessed *FGFR1* amplification, one patient with esophageal cancer and *FGFR3* translocation, one patient with urothelial tract/bladder cancer and *FGFR3* mutation (p.Y373C), one patient with breast cancer and *FGFR2* amplification, and one patient with “other” (rectal cancer) and *FGFR1* amplification.

## 4 DISCUSSION

In this study, no DLTs were observed and the MTD of pemigatinib was not reached. Safety, tolerability, and PK data supported the selection of 13.5 mg as the RP2D and this was the starting dose for Part 2 of this study. The safety, and PK and pharmacodynamics profiles of pemigatinib in Japanese patients with advanced solid tumors are similar to that observed in patients in the United States and Denmark in the FIGHT‐101 study.

The most common (>30%) TEAEs were consistent with on‐target effects of FGFR pathway inhibition and/or conditions associated with underlying disease, and included hyperphosphatemia, dysgeusia, stomatitis, alopecia, decreased appetite, diarrhea, nausea, and constipation. In general, the incidence of any grade TEAEs was higher among patients receiving CD compared with ID schedules. These results are consistent with results reported in the FIGHT‐101 study in patients from the United States and Denmark. The most common (>30%) overall TEAEs in FIGHT‐101 were hyperphosphatemia, fatigue, dry mouth, stomatitis, diarrhea, and alopecia, with a higher incidence of TEAEs overall among patients on CD regimen compared with an ID regimen.^11^

Hyperphosphatemia is an anticipated on‐target pharmacologic effect of FGFR inhibition.^20^ The study protocol recommended management of hyperphosphatemia by dietary phosphate restriction, administration of phosphate‐lowering therapy, and pemigatinib dose modifications. Notably, half of patients in the study received lanthanum carbonate, a phosphate‐binding agent to help these patients maintain healthy phosphate concentrations. Importantly, no event of hyperphosphatemia was serious or led to pemigatinib discontinuation. Hypophosphatemia was reported in two patients; both events were Grades 1 or 2 in severity.

Other events of clinical interest associated with selective FGFR inhibitors include nail and ocular toxicities, including serous retinal detachment.^21^ In this study, AEs related to eye disorders were generally mild to moderate, self‐limiting, or manageable with dose modification. None of the nail toxicity events were Grade ≥3. None of the clinically notable TEAEs led to pemigatinib discontinuation.

Phase 1 studies of other FGFR inhibitors in Japanese patients have reported similar AE profiles as reported in our study.^22^, ^23^, ^24^ Consistent with our findings, phase 1 studies of futibatinib and erdafitinib in Japanese patients with advanced solid tumors have reported hyperphosphatemia as the most common TEAE.^22^, ^23^, ^24^ Other TEAEs, including stomatitis, dysgeusia, nausea, diarrhea and retinal detachment, were also reported in these studies.^23^, ^24^

In a global phase 1 study of infigratinib, a selective FGFR1‐3 inhibitor, the most commonly reported (>30%) TEAEs were hyperphosphatemia, constipation, appetite decreased, stomatitis, diarrhea, nausea, and fatigue. Other toxicities associated with selective FGFR pathway inhibition such as eye‐related disorders were also reported in infigratinib‐treated patients.^25^ In a phase 1 multicenter study of erdafitinib in patients with advanced solid tumors, the most common TEAEs were hyperphosphatemia, dry mouth, stomatitis, and asthenia.^26^ Similarly, in a recent report of a phase 1 study of futibatinib in patients with advanced solid tumors, the most frequently reported TEAEs were hyperphosphatemia, diarrhea, constipation, and nausea.^27^

Following a single dose of pemigatinib 9 or 13.5 mg QD, plasma concentrations of pemigatinib remained at or above the in vivo IC_50_ for the inhibition of pFGFR2.^10^ The steady‐state *C*_max_ geometric means were 195 nM and 236 nM, and AUC_0‐24h_ geometric means were 2720 h × nM and 2620 h × nM between Japanese patients in this study and patients in the United States and Denmark in the phase 1 FIGHT‐101 study.

Plasma FGF23 was used as a prespecified marker for *FGFR* activity. FGF23 plays an important role in phosphate homeostasis and is induced in FGFR inhibitor rodent models.^28^ In this study, pemigatinib induced an increase in plasma *FGF23* concentration from baseline to Day 15 in 90% of patients. For patients receiving 13.5 mg pemigatinib QD, mean plasma FGF23 concentration approximately doubled from baseline in the initial 2 weeks of treatment and did not change statistically significantly thereafter. Ex vivo pharmacodynamic analysis showed 80% mean inhibition of pFGFR2α at trough pemigatinib concentration (C1D15, 0 h) at all dosages evaluated. These results confirm the biologic activity of pemigatinib in Japanese patients with solid tumors.

Efficacy analyses demonstrated clinical activity with five PRs reported across tumors in patients receiving pemigatinib 13.5 mg QD in Part 2 including one receiving ID and four receiving CD. All of the five patients had *FGF/FGFR* alterations. Four patients with cholangiocarcinoma had SD, three of whom had *FGFR2* alterations.

This study had limitations that are common for phase 1/2 studies. No statistical comparisons across dosing regimens or cancer types were planned. Therefore, the study was not sufficiently powered for such comparisons. Only a small number of patients with a variety of solid tumors were enrolled.

## 5 CONCLUSION

In this study of pemigatinib in Japanese patients with advanced solid tumors, no new safety signals were observed. Adverse events were typical of those seen with FGFR inhibition and were generally manageable. The safety, PK, and pharmacodynamic profiles of pemigatinib in Japanese patients with advanced solid tumors in this study are consistent with those observed previously in FIGHT‐101.^11^ Clinical responses were observed with five PRs across different tumor types including cholangiocarcinoma with *FGFR2* alterations.

## AUTHOR CONTRIBUTIONS

**Yutaka Fujiwara:** Investigation (equal); visualization (equal); writing – review and editing (equal). **Yasutoshi Kuboki:** Investigation (equal); writing – original draft (equal); writing – review and editing (equal). **Masayuki Furukawa:** Investigation (equal); writing – review and editing (equal). **Nobumasa Mizuno:** Investigation (equal); writing – review and editing (equal). **Hiroki Hara:** Investigation (equal); writing – review and editing (equal). **Tatsuya Ioka:** Investigation (equal); writing – review and editing (equal). **Makoto Ueno:** Investigation (equal); writing – review and editing (equal). **Yasuo Takahashi:** Investigation (equal); writing – review and editing (equal). **Shunji Takahashi:** Investigation (equal); writing – review and editing (equal). **Shinji Takeuchi:** Investigation (equal); writing – review and editing (equal). **Christine Lihou:** Conceptualization (equal); data curation (equal); formal analysis (equal); investigation (equal); methodology (equal); project administration (equal); supervision (equal); writing – original draft (equal); writing – review and editing (equal). **Tao Ji:** Formal analysis (equal); methodology (equal); software (equal). **Chenwei Tian:** Formal analysis (equal); methodology (equal); validation (equal). **Toshio Shimizu:** Investigation (equal); writing – review and editing (equal).

## CONFLICT OF INTEREST STATEMENT

Yutaka Fujiwara: personal fees from Amgen, AstraZeneca, Bristol Myers Squibb, Chiome Bioscience, Chugai Pharmaceutical, Daiichi Sankyo, Eli Lilly, Novartis, ONO Pharmaceutical, Otsuka Pharmaceutical, Pfizer, Taiho, Takeda, and Yakult, and grants from AnHeart Therapeutics and MSD outside the submitted work; Yasutoshi Kuboki: grants and personal fees from AbbVie, Amgen, Astellas, AstraZeneca, Boehringer Ingelheim, Bristol Myers Squibb, Chugai, Daiichi‐Sankyo, Genmab, GlaxoSmithKline, Incyte, Lilly, Ono, Taiho, and Takeda; Masayuki Furukawa: nothing to disclose; Nobumasa Mizuno: grants from Incyte during the conduct of the study; and grants and personal fees from ASLAN Pharmaceuticals, AstraZeneca, Eisai, FUJIFILM Toyama Chemical, MSD, NanoCarrier, Novartis, Ono Pharmaceutical, Seagen, Taiho Pharmaceutical, and Yakult Honsha; Hara Hiroki: grants or contracts from Amgen, Astellas, AstraZeneca, Bayel, BeiGene, Boehringer Ingelheim, Chugai, Daiichi Sankyo, Dainippon Sumitomo, Janssen, Merck Biopharma, MSD, Ono Pharmaceutical, and Taiho; consulting fees from Boehringer Ingelheim, Bristol Myers Squibb, Daiichi Sankyo, Dainippon Sumitomo, MSD, Ono Pharmaceutical; payment or honoraria for lectures, presentations, speakers' bureaus, manuscript writing or educational events from Asahi Kasei, Bayer, Bristol Myers Squibb, Chugai, Daiichi Sankyo, Kyowa Hakko Kirin, Lilly, Merck Biopharma, MSD, Ono Pharmaceutical, Sanofi, Taiho, Takeda, and Yakult; Tatsuya Ioka: personal fees from Incyte, Ono Pharmaceutical, and Taiho Pharmaceutical outside the submitted work; Makoto Ueno: grants and personal fees from Astellas Pharma, AstraZeneca, Chugai Pharmaceuticals, DFP, Eisai, Incyte, Merck Biopharma, MSD, Nihon Servier, Ono Pharmaceutical, and Taiho Pharmaceutical; Yasuo Takahashi: Nothing to disclose; Shunji Takahashi: grants from Incyte during the conduct of the study; grants and personal fees from AstraZeneca, Bayer, Bristol Myers Squibb, Chugai, DAIICHI‐SANKYO, Eisai, MSD, Novartis, Ono pharmaceutical, and TAIHO, outside the submitted work; Shinji Takeuchi: grants and personal fees from Array BioPharma, Chugai, CMIC, Eli Lilly, Loxo Oncology, Ono, Pfizer, and Takeda; Chenwei Tian: employee and shareholder of Incyte Corporation; Christine Lihou and Tao Ji: former employees and shareholders of Incyte Corporation; Toshio Shimizu: grants from Incyte during the conduct of the study; grants and personal fees from 3D‐Medicine, AbbVie, Astellas, AstraZeneca, Bristol Myers Squibb, Chordia Therapeutics, Daiichi‐Sankyo, Eisai, LOXO/Eli Lilly, Novartis, Pfizer, PharmaMar, Symbio Pharmaceuticals, and Takeda Oncology, outside the submitted work.

## Supporting information


Data S1.

## Data Availability

Incyte Corporation (Wilmington, DE) is committed to data sharing that advances science and medicine while protecting patient privacy. Qualified external scientific researchers may request anonymized datasets owned by Incyte for the purpose of conducting legitimate scientific research. Researchers may request anonymized datasets from any interventional study (except phase 1 studies) for which the product and indication have been approved on or after January 1, 2020 in at least one major market (e.g., United States, European Union, Japan). Data will be available for request after the primary publication or 2 years after the study has ended. Information on Incyte's clinical trial data sharing policy and instructions for submitting clinical trial data requests are available at: https://www.incyte.com/Portals/0/Assets/Compliance%20and%20Transparency/clinical‐trial‐data‐sharing.pdf?ver=2020‐05‐21‐132838‐960.
