# Supplementary material for: FIGHT‐102: A phase 1 study of pemigatinib in Japanese patients with advanced malignancies
Source: Cancer Med. 2023 Mar 31;12(9):10597–611. doi: 10.1002/cam4.5798 (PMC10225205; doi:10.1002/cam4.5798)

# SUPPLEMENTARY MATERIAL

# FIGHT-102: A phase 1 study of pemigatinib in Japanese patients with advanced malignancies

Yukata Fujiwara,[1,2] Yasutoshi Kuboki,[3*] Masayuki Furukawa,[4] Nobumasa Mizuno,[2] Hiroki Hara,[5] Tatsuya Ioka,[6] Makoto Ueno,[7] Yasuo Takahashi,[8] Shunji Takahashi,[9] Shinji Takeuchi,[10] Christine Lihou,[11] Tao Ji,[11] Chenwei Tian,[11] Toshio Shimizu[1,12]

**Affiliations**

[1]National Cancer Center Hospital, Chuo-ku, Tokyo, Japan; [2]Aichi Cancer Center, Chikusa-ku, Nagoya, Japan; [3]National Cancer Central Hospital East, Kashiwa, Chiba, Japan; [4]Kyushu Cancer Center, Minami-ku, Fukuoka, Japan; [5]Saitama Cancer Center, Saitama, Japan; [6]Department of Oncology Center, Yamaguchi University Hospital, Ube, Japan; [7]Kanagawa Cancer Center, Yokohama, Kanagawa, Japan; [8]Hokkaido Cancer Center, Sapporo, Japan; [9]Cancer Institute Hospital of JFCR, Ariake, Koto-ku, Tokyo, Japan; [10]Kanazawa University Hospital, Japan; [11]Incyte Corporation, Wilmington, DE, USA; [12]Wakayama Medical University, Wakayama, Japan

***Correspondence:**

Prof. Yasutoshi Kuboki, MD, PhD

National Cancer Central Hospital East

6-5-1 Kashiwanoha, Kashiwa-shi

Chiba, 277-8577

Japan

Email: ykuboki@east.ncc.go.jp

# Supplementary Tables

## **Supplementary Table 1.** *FGF/FGFR* status at baseline

| **Centrally confirmed *FGF/FGFR* alterations at baseline[a,b], n (%)** | **Pemigatinib intermittent dosing** | | | | **Pemigatinib continuous dosing** | |
|---|---|---|---|---|---|---|
| | **9 mg (n = 3)** | **13.5 mg (n = 23)** | **18 mg (n = 5)** | **Subtotal (n = 31)** | **13.5 mg (n = 13)** | **Total (n = 44)** |
| *FGFR1* amplification | 0 | 8 (34.8) | 0 | 8 (25.8) | 3 (23.1) | 11 (25.0) |
| *FGFR 2* amplification | 0 | 2 (8.7) | 0 | 2 (6.5) | 1 (7.7) | 3 (6.8) |
| *FGFR2* translocation | 0 | 1 (4.3) | 0 | 1 (3.2) | 0 | 1 (2.3) |
| *FGFR3* alteration | 0 | 0 | 0 | 0 | 1 (7.7) | 1 (2.3) |
| *FGFR3* mutation | 0 | 2 (8.7) | 0 | 2 (6.5) | 0 | 2 (4.5) |
| *FGFR3* translocation | 0 | 0 | 0 | 0 | 1 (7.7) | 1 (2.3) |
| *FGF1* amplification | 0 | 1 (4.3) | 0 | 1 (3.2) | 0 | 1 (2.3) |
| *FGF3* amplification | 0 | 6 (26.1) | 0 | 6 (19.4) | 3 (23.1) | 9 (20.5) |
| *FGF4* amplification | 0 | 5 (21.7) | 0 | 5 (16.1) | 3 (23.1) | 8 (18.2) |
| *FGF6* mutation | 0 | 1 (4.3) | 0 | 1 (3.2) | 0 | 1 (2.3) |
| *FGF12* amplification | 0 | 2 (8.7) | 0 | 2 (6.5) | 0 | 2 (4.5) |
| *FGF19* amplification | 0 | 5 (21.7) | 0 | 5 (16.1) | 3 (23.1) | 8 (18.2) |

FGF, fibroblast growth factor; FGFR, fibroblast growth factor receptor. [a]Locally identified *FGF/FGFR* alterations are not presented in the table.
[b]Some patients had more than 1 relevant alteration.

**Supplementary Table 2.** Treatment-related adverse events[a]

| Number of events (%) | Pemigatinib intermittent dosing | | | | | | | | Pemigatinib continuous dosing | | Total (N = 44) | |
|---|---|---|---|---|---|---|---|---|---|---|---|---|
| | 9 mg (n = 3) | | 13.5 mg (n = 23) | | 18 mg (n = 5) | | Subtotal (n = 31) | | 13.5 mg (n = 13) | | | |
| | All grades | Grade ≥3 | All grades | Grade ≥3 | All grades | Grade ≥3 | All grades | Grade ≥3 | All grades | Grade ≥3 | All grades | Grade ≥3 |
| Hyperphosphatemia | 2 (66.7) | 0 | 18 (78.3) | 0 | 5 (100.0) | 0 | 25 (80.6) | 0 | 11 (84.6) | 0 | 36 (81.8) | 0 |
| Dysgeusia | 1 (33.3) | 0 | 7 (30.4) | 0 | 2 (40.0) | 0 | 10 (32.3) | 0 | 8 (61.5) | 0 | 18 (40.9) | 0 |
| Stomatitis | 1 (33.3) | 0 | 3 (13.0) | 0 | 4 (80.0) | 0 | 8 (25.8) | 0 | 10 (76.9) | 1 (7.7) | 18 (40.9) | 1 (2.3) |
| Alopecia | 1 (33.3) | 0 | 7 (30.4) | 0 | 2 (40.0) | 0 | 10 (32.3) | 0 | 7 (53.8) | 0 | 17 (38.6) | 0 |
| Nausea | 0 | 0 | 7 (30.4) | 0 | 3 (60.0) | 0 | 10 (32.3) | 0 | 4 (30.8) | 0 | 14 (31.8) | 0 |
| Diarrhea | 1 (33.3) | 0 | 4 (17.4) | 0 | 1 (20.0) | 0 | 6 (19.4) | 0 | 7 (53.8) | 1 (7.7) | 13 (29.5) | 1 (2.3) |
| Decreased appetite | 0 | 0 | 4 (17.4) | 0 | 3 (60.0) | 2 (40.0) | 7 (22.6) | 0 | 4 (30.8) | 1 (7.7) | 11 (25.0) | 1 (2.3) |
| Blood creatinine increased | 0 | 0 | 4 (17.4) | 0 | 1 (20.0) | 0 | 5 (16.1) | 0 | 1 (7.7) | 0 | 6 (13.6) | 0 |
| Fatigue | 0 | 0 | 2 (8.7) | 0 | 1 (20.0) | 0 | 3 (9.7) | 0 | 3 (23.1) | 0 | 6 (13.6) | 0 |
| Paronychia | 0 | 0 | 2 (8.7) | 0 | 0 | 0 | 2 (6.5) | 0 | 4 (30.8) | 0 | 6 (13.6) | 0 |
| Alanine aminotransferase increased | 1 (33.3) | 0 | 2 (8.7) | 0 | 0 | 0 | 3 (9.7) | 0 | 2 (15.4) | 1 (7.7) | 5 (11.4) | 1 (2.3) |
| Arthralgia | 0 | 0 | 2 (8.7) | 0 | 0 | 0 | 2 (6.5) | 0 | 3 (23.1) | 0 | 5 (11.4) | 0 |

| | | | | | | | | | | | |
|---|---|---|---|---|---|---|---|---|---|---|---|
| Aspartate aminotransferase increased | 0 | 0 | 3 (13.0) | 0 | 0 | 0 | 3 (9.7) | 0 | 2 (15.4) | 1 (7.7) | 5 (11.4) | 1 (2.3) |
| Dry mouth | 0 | 0 | 1 (4.3) | 0 | 0 | 0 | 1 (3.2) | 0 | 4 (30.8) | 0 | 5 (11.4) | 0 |
| Keratitis | 1 (33.3) | 0 | 2 (8.7) | 0 | 0 | 0 | 3 (9.7) | 0 | 2 (15.4) | 0 | 5 (11.4) | 0 |
| Malaise | 0 | 0 | 2 (8.7) | 0 | 0 | 0 | 2 (6.5) | 0 | 3 (23.1) | 0 | 5 (11.4) | 0 |
| Onchyomadesis | 0 | 0 | 2 (8.7) | 0 | 0 | 0 | 2 (6.5) | 0 | 3 (23.1) | 0 | 5 (11.4) | 0 |
| Palmar-plantar erythrodysesthesia syndrome | 0 | 0 | 2 (8.7) | 0 | 0 | 0 | 2 (6.5) | 0 | 3 (23.1) | 0 | 5 (11.4) | 0 |
| Serous retinal detachment | 1 (33.3) | 0 | 3 (13.0) | 0 | 0 | 0 | 4 (12.9) | 0 | 1 (7.7) | 0 | 5 (11.4) | 0 |

[a]Reported in ≥10% of patients.

**Supplementary Table 3.** Pharmacokinetic parameters on Cycle 1 Day 1

| Dose | Patients (n) | $C_{max}$ (nM) | $t_{max}$ (h) | $AUC_{last}$ (h*nM) | $AUC_{0\text{-}24h}$ (h*nM) |
|---|---|---|---|---|---|
| 9 mg ID | 3 | 114 ± 35.3<br>110 (32.1) | 1.90<br>(0.783–3.95) | 1120 ± 125<br>1120 (11.6) | 1130 ± 133<br>1120 (12.2) |
| 13.5 mg ID orCD | 36 | 260 ± 136<br>216 (77.0) | 1.43<br>(0.500–6.00) | 1990 ± 748<br>1850 (41.2) | 2020 ± 762<br>1880 (41.2) |
| 18 mg ID | 5 | 431 ± 272<br>344 (96.7) | 1.02<br>(0.467–6.02) | 3380 ± 1010<br>3240 (34.3) | 3450 ± 1020<br>3310 (33.7) |

Note: Values are presented as mean ± SD and geometric mean (CV%), except $t_{max,}$ which is reported as median (range).
$AUC_{0\text{-}24h}$, area under the plasma concentration-time curve from hours 0 to 24; $AUC_{last}$, area under the plasma concentration-time curve from time 0 to the last measurable concentration; CD, continuous dosing; $C_{max}$, maximum plasma drug concentration; ID, intermittent dosing; $t_{max}$, time to maximum plasma drug concentration.

**Supplementary Table 4.** Pharmacokinetic parameters at steady state (Cycle 1 Day 14)

| **Dose** | **Patients (n)** | **$C_{max, ss}$ (nM)** | **$t_{max}$ (h)** | **$t_{½}$ (h)** | **$C_{min, ss}$ (nM)** | **$AUC_{ss,0-24h}$ (h*nM)** | **$CL_{ss/F}$ (L/h)** | **$V_z$/F (L)** | **Accumulation ratio** |
|---|---|---|---|---|---|---|---|---|---|
| 9 mg ID | 3 | 155 ± 117<br>127 (89.9) | 0.967<br>(0.917–1.00) | 17.1 ± 7.44<br>16.1 (43.6) | 74.7 ± 60.0<br>60.4 (92.9) | 2540 ± 2100<br>2050 (91.0) | 10.6 ± 6.31<br>8.99 (91.0) | 224 ± 104<br>209 (48.1) | 2.43 ± 2.29<br>1.83 (111) |
| 13.5 mg ID or CD | 33 | 238 ± 139<br>195 (77.8) | 1.02<br>(0.750–24.0) | 15.4 ± 9.03<br>13.6 (49.2) | 66.2 ± 45.2<br>56.0 (61.0) | 3090 ± 1680<br>2720 (55.5) | 11.6 ± 6.52<br>10.2 (55.5) | 258 ± 216<br>201 (76.9) | 1.59 ± 0.767<br>1.43 (52.3) |
| 18 mg ID | 3 | 450 ± 285<br>385 (81.1) | 0.833<br>(0.000–1.00) | 20.7 ± 11.2<br>18.9 (54.3) | 203 ± 52.1<br>199 (24.7) | 6950 ± 2780<br>6560 (43.8) | 5.96 ± 2.50<br>5.63 (43.8) | 205 ± 188<br>154 (116) | 1.86 ± 0.456<br>1.83 (23.5) |

Note: Values are presented as mean ± SD and geometric mean (CV%), except $t_{max}$ which is reported as median (range).

$AUC_{SS,0-24}$, area under the plasma concentration-time curve from hours 0 to 24 during steady state; CD, continuous dosing; $CL_{ss}$/F, apparent oral dose clearance at steady state; $C_{max,ss}$, maximum plasma drug concentration at steady state; $C_{min,ss}$, minimum plasma drug concentration at steady state; CV, coefficient of variation; ID, intermittent dosing; QD, once daily; SD, standard deviation; $t_{max}$, time to maximum plasma drug concentration; $t_{½}$, half-life; $V_z$/F, apparent oral volume of distribution.

**Supplementary Table 5.** Efficacy results by genomic alteration

| Dose regimen | Solid tumor type | Genomic alteration | | Best change in sum of target lesion diameters (%) | Best overall response |
|---|---|---|---|---|---|
| | | Central laboratory | Local laboratory | | |
| **Part 1** | | | | | |
| 9 mg ID | Gastric cancer | Not tested | Not tested | –13.3 | SD |
| 9 mg ID | Other (medulloblastoma) | Not tested | No alteration detected | –[a] | PD |
| 9 mg ID | Other (epithelioid hemangioendothelioma) | Not tested | Not tested | –12.6 | SD |
| 13.5 mg ID | Other (intrahepatic cholangiocarcinoma) | No alteration detected | Not tested | 4.4 | SD |
| 13.5 mg ID | Cholangiocarcinoma | *FGF1* amp, *FGFR2* amp | Not tested | –8.5 | SD |
| 13.5 mg ID | Other (lower bile duct cancer) | *FGF6* mutation (p.S143N) | Not tested | –5.1 | SD |
| 13.5 mg ID | Colorectal cancer | *FGFR1* amp | *FGFR1* amp | –21.1 | PD |
| 13.5 mg ID | Lung cancer (NSCLC) | Not tested | Not tested | –29.0 | SD |
| 13.5 mg ID | Breast cancer | Not tested | No alteration detected | 28.1 | PD |
| 18 mg ID | Other (intrahepatic cholangiocarcinoma) | No alteration detected | *FGFR2* fusion | 0 | SD |
| 18 mg ID | Other (intrahepatic cholangiocarcinoma) | Not tested | Not tested | 16.7 | PD |
| 18 mg ID | Other (Pancreatic head cancer) | Not tested | No alteration detected | 1.0 | PD |
| 18 mg ID | Other (oropharyngeal cancer) | No alteration detected | Not tested | –[a] | –[a] |

| | | | | | |
|---|---|---|---|---|---|
| 18 mg ID | Neuroendocrine cancer | Not tested | *FGF6/23* amp | 37.5 | PD |
| **Part 2** | | | | | |
| 13.5 mg ID | Cholangiocarcinoma | *FGFR3* mutation | Not tested | 30.9 | PD |
| 13.5 mg ID | Gallbladder cancer | No alteration detected | *FGFR3* mutation | 25.0 | PD |
| 13.5 mg ID | Cholangiocarcinoma | *FGFR2* alteration | *FGFR2* translocation | –13.8 | SD |
| 13.5 mg ID | Other (intrahepatic cholangiocarcinoma) | *FGFR3* fusion | Not tested | 65.2 | PD |
| 13.5 mg ID | Colorectal cancer | *FGFR1* amp | Not tested | 44.7 | PD |
| 13.5 mg ID | Esophageal cancer | *FGFR1* amp | Not tested | 6.5 | SD |
| 13.5 mg ID | Esophageal cancer | *FGF3/4/12/19* amp | Not tested | 34.9 | PD |
| 13.5 mg ID | Esophageal cancer | *FGFR1* amp, *FGF3/4/19* amp | Not tested | 37.8 | PD |
| 13.5 mg ID | Esophageal cancer | *FGFR1* amp, *FGF3/4/19* amp | Not tested | 3.0 | PD |
| 13.5 mg ID | Esophageal cancer | *FGF3/4/19* amp | *FGF19* amp | –[a] | PD |
| 13.5 mg ID | Breast cancer | *FGFR1* amp | Not tested | 18.1 | PD |
| 13.5 mg ID | Urothelial tract/ bladder cancer (renal pelvis cancer) | *FGF3/4/19* amp | Not tested | 24.2 | PD |
| 13.5 mg ID | Other (urachal cancer) | *FGF3* amp | Not tested | 44.8 | PD |
| 13.5 mg ID | Pancreatic cancer (neuroendocrine) | *FGFR1* amp | *FGFR1* mutation (p.N577K) | 40.8 | PD |
| 13.5 mg ID | Lung cancer (NSCLC) | *FGFR1* amp, *FGF12* amp | Not tested | 22.2 | PD |

| | | | | | |
|---|---|---|---|---|---|
| 13.5 mg ID | Other (apocrine sweat gland carcinoma) | *FGFR2* amp | Not tested | –42.1 | PR |
| 13.5 mg ID | Urothelial tract/ bladder cancer (bladder cancer) | *FGFR3* mutation (p.Y373C) | Not tested | –19.6 | SD |
| 13.5 mg CD | Cholangiocarcinoma | No alteration detected | *FGFR2* translocation | –30.6 | PR |
| 13.5 mg CD | Gallbladder cancer | *FGF3/4/19* amp | Not tested | –43.8 | PR |
| 13.5 mg CD | Colorectal cancer | No alteration detected | *FGF19* amp | 41.0 | PD |
| 13.5 mg CD | Colorectal cancer | *FGFR1* amp | Not tested | 26.5 | PD |
| 13.5 mg CD | Breast cancer | Not tested | *FGFR2* amp | –26.2 | SD |
| 13.5 mg CD | Breast cancer | Not tested | *FGFR1* amp, *FGFR3* mutation | 6.4 | NE |
| 13.5 mg CD | Breast cancer | Not tested | *FGFR2* amp | –44.4 | PR |
| 13.5 mg CD | Urothelial tract/ bladder cancer (bladder cancer) | *FGF3/4/19* amp*, FGFR3 alteration* | Not tested | –37.2 | PR |
| 13.5 mg CD | Other (rectal cancer) | *FGFR1* amp | Not tested | 0.0 | SD |
| 13.5 mg CD | Other (uterine cervix carcinoma) | *FGFR2* amp | Not tested | 22.7 | PD |
| 13.5 mg CD | Other (prostate cancer) | *FGFR1* amp | Not tested | 15.2 | PD |
| 13.5 mg CD | Urothelial tract/ bladder cancer (renal pelvis cancer) | *FGF3/4/19* amp | Not tested | 12.1 | PD |
| 13.5 mg CD | Esophageal cancer | *FGFR3* translocation: fusion | *FGFR3* translocation: fusion | –4.3 | SD |

[a] Missing baseline and/or postbaseline disease assessment.

amp, amplification; CD, continuous dosing; FGFR, fibroblast growth factor receptor; ID, intermittent dosing; NE, not evaluable; NSCLC, non-small cell lung cancer; PD, progressive disease; PR, partial response; SD, stable disease.

## Supplementary Figures

**Supplementary Figure 1.** FIGHT-102 study design. FGF, fibroblast growth factor; FGFR, fibroblast growth factor receptor; LDL, lower dose level; PK, pharmacokinetics; QD, once daily; RP2D, recommended phase 2 dose.

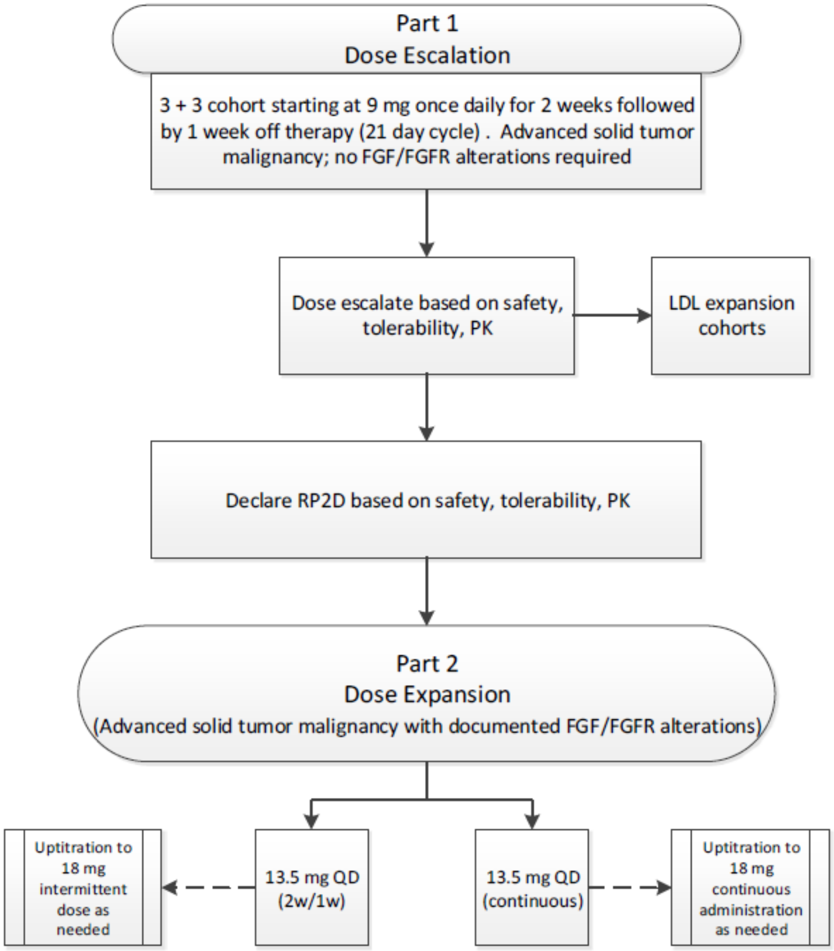

**Supplementary Figure 2.** Comparison of pemigatinib exposures at 13.5 mg once daily in FIGHT-101 and FIGHT-102. Data are presented as mean ± SD and geometric mean (CV%). [a]In FIGHT-101, 50 and 29 patients received 13.5 mg QD in the ID and CD regimens, respectively. [b]In FIGHT-102, 23 and 13 patients received 13.5 mg QD in the ID and CD regimens, respectively. $AUC_{ss}$,0–24, area under the plasma concentration-time curve from hour 0 to 24 at steady state; CD, continuous dosing; $C_{max, ss}$, maximum plasma drug concentration at steady state; CV, coefficient of variation; h, hour; ID, intermittent dosing; nM, nanomolar; SD, standard deviation.

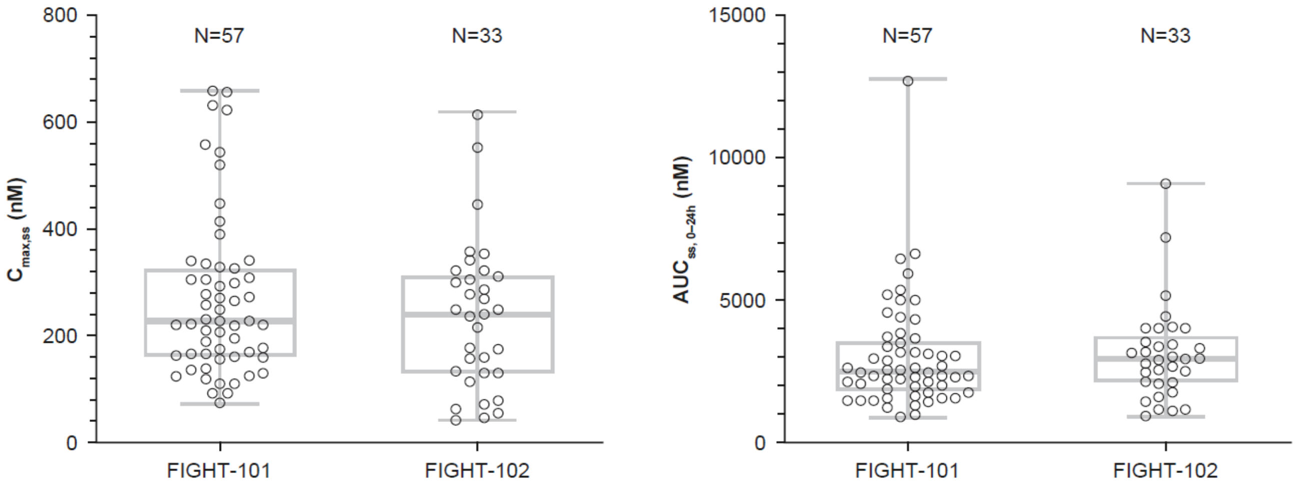


| Population | Patients (n) | $C_{max,ss}$ (nM) | $AUC_{ss, 0–24h}$ (h*nM) |
|---|---|---|---|
| FIGHT-101 | 57 | 271 ± 151<br>236 (56.4) | 3010 ± 1890<br>2620 (54.1) |
| FIGHT-102 | 33 | 238 ± 139<br>195 (77.8) | 3090 ± 1680<br>2720 (55.5) |

**Supplementary Figure 3.** Induction of plasma *FGF23* after treatment with pemigatinib 13.5 mg. Fold changes from baseline in *FGF23* expression were plotted for individual patients receiving pemigatinib 13.5-mg on ID or CD schedules. Boxes denote the first and third quartiles, lines represent the median, and the whiskers show the limits of 1.5 × IQR. Dashed grey line denotes no fold change from baseline. C, cycle; CD, continuous dosing; D, day; *FGF23*, fibroblast growth factor 23; ID, intermittent dosing; IQR, interquartile range.

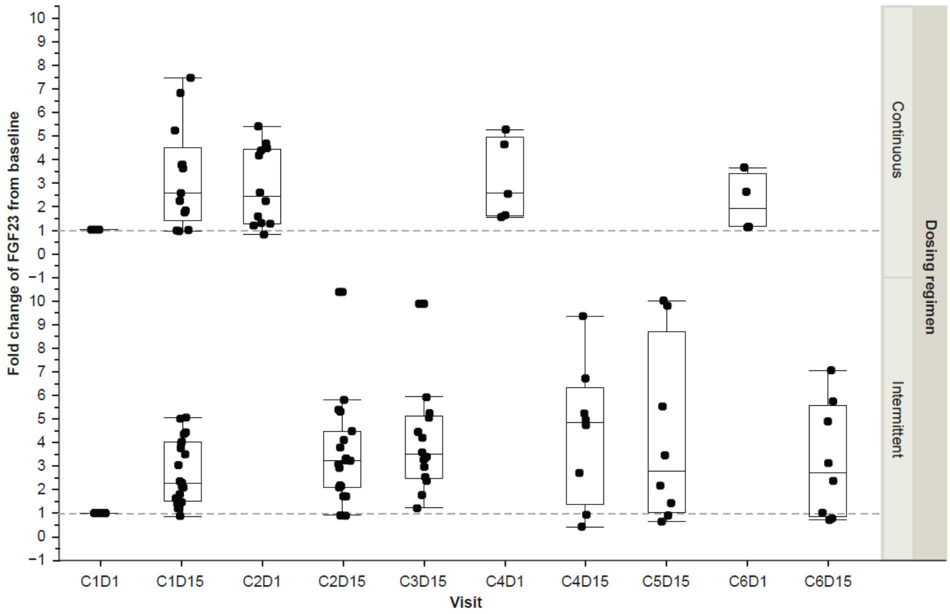

**Supplementary Figure 4.** Inhibition of FGFR2α phosphorylation at trough pemigatinib concentration (Cycle 1 Day 15, 0 hours) in the KATO III *ex vivo* assay. Data are presented as mean ± SD. Dashed line shows 80% inhibition of FGFR2α phosphorylation. FGFR2α, fibroblast growth factor receptor 2-alpha; SD, standard deviation.

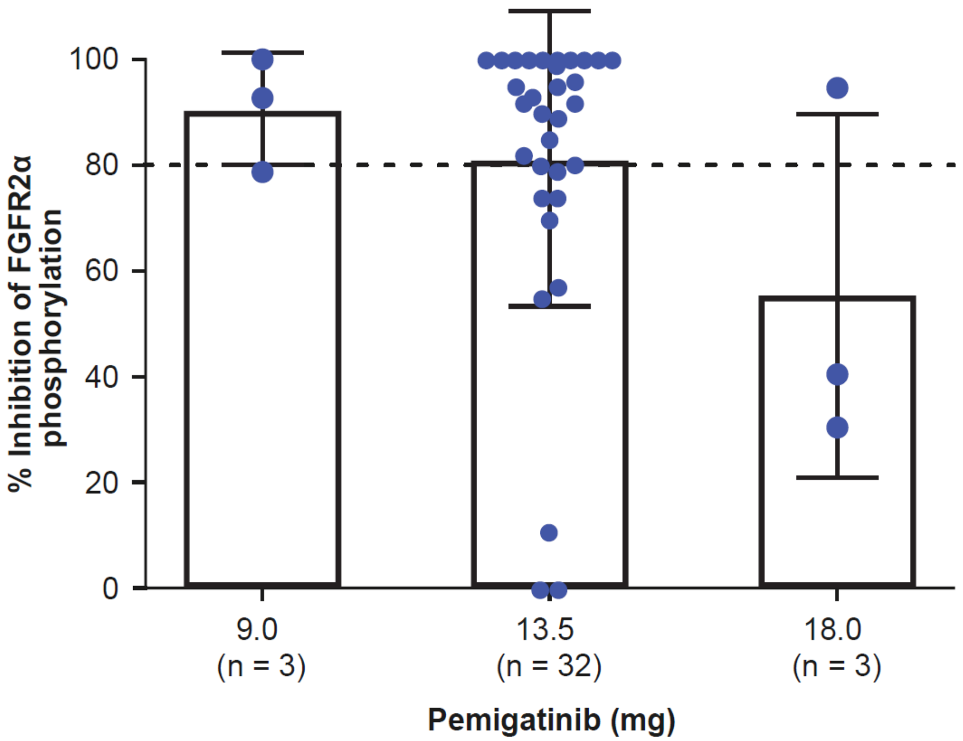

Supplement: Supplementary file 1 — Data S1. [file CAM4-12-10597-s001.pdf]
